# Radiofrequency wire-assisted recanalization of a chronically occluded common femoral vein after intravenous drug use

**DOI:** 10.1016/j.jvscit.2025.102091

**Published:** 2025-12-12

**Authors:** Kathleen Gibson, Sooyeon Kim, Kush Desai

**Affiliations:** a Overlake Vascular Surgeons, Bellevue, WA; b Department of Interventional Radiology, Northwestern University Medicine, Chicago, IL

**Keywords:** Venous occlusion, IV drug use, Venous recanalization

## Abstract

Chronic common femoral vein occlusion presents a significant management challenge, particularly when associated with prior intravenous drug use. We report a 52-year-old woman, sober for 10 years, with symptomatic chronic common femoral vein occlusion after two failed recanalization attempts. Successful recanalization was achieved with a Baylis radiofrequency wire (Baylis Medical), followed by balloon venoplasty and placement of a 14-mm Abre venous stent (Medtronic). The patient experienced symptomatic improvement with preserved patency at 3 months. To our knowledge, this report is the first to describe radiofrequency wire-assisted crossing of a chronic common femoral vein occlusion in a patient with a history of intravenous drug use. We also highlight technique considerations unique to the femoral region—multiplanar venography and the use of arterial landmarks to avoid the femoral artery and femoral nerve—to enhance procedural safety.

Chronic iliofemoral venous obstruction can cause pain, swelling, venous claudication, and functional limitation. Prior intravenous drug use (IVDU) causes significant venous wall injury that predisposes to acute thrombosis and chronic fibrotic occlusion, which in our experience can be more resistant to recanalization.^1^, ^2^, ^3^, ^4^ Although many patients achieve long-term sobriety, the consequences of resultant venous hypertension may persist, leading to morbidity despite conservative care.

Endovascular therapy is the mainstay for symptomatic chronic venous occlusions. Standard wire catheter techniques may traverse many lesions, but heavily fibrotic occlusions can resist both conventional and sharp (ie, needle-based) recanalization. In such cases, alternative tools are required.

The Baylis radiofrequency (RF) wire has been used in select refractory iliocaval venous occlusions, offering controlled energy delivery to cross fibrotic occlusions under fluoroscopic guidance. We present a 52-year-old woman with prior IVDU and chronic common femoral vein occlusion successfully recanalized with an RF wire after multiple failed prior attempts. The patient provided written informed consent for publication of this case report and accompanying images.

## Case report

A 52-year-old woman with prior IVDU (sober 10 years) had severe daily heaviness, aching, swelling, and throbbing, leading to limitation in sitting, standing, and walking. Despite long-term compression, elevation, weight loss, and exercise, symptoms worsened, and work became physically difficult.

Duplex ultrasound examination showed bilateral superficial venous reflux and left common femoral vein occlusion. Computed tomographic venography confirmed caudal external iliac occlusion. Two prior endovascular attempts failed despite sharp techniques (back end of a stiff Glidewire [Terumo], Rösch-Uchida needle [Cook], and Beback crossing catheter [Cook]). As is our standard practice, off-label device use was explicitly reviewed with the patient during the informed consent process. She demonstrated understanding of the rationale, risks, and alternatives and elected to proceed given that her symptoms were significantly limiting both ambulation and her physically demanding work.

**Fig 1:**
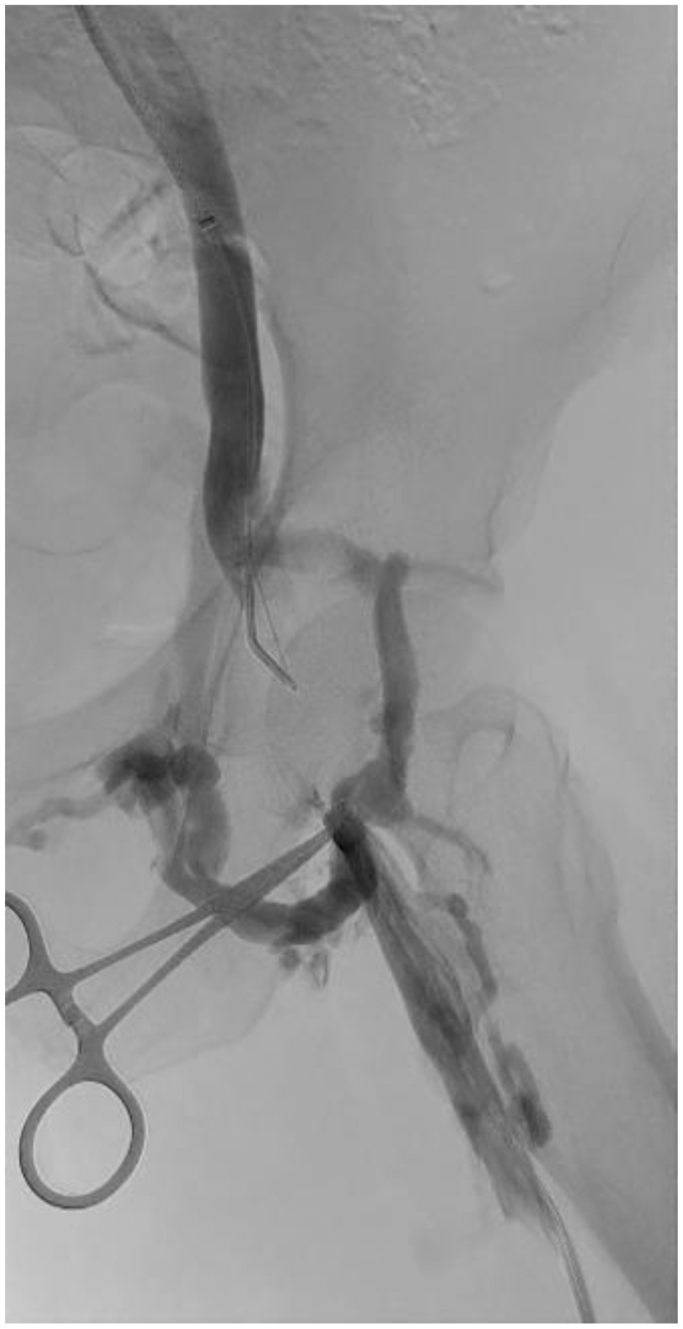
Chronic occlusion of the left common femoral vein with reconstitution of the external iliac vein.

Under general anesthesia, ultrasound-guided access was obtained in the left femoral vein (9F) in the mid to upper thigh and right internal jugular vein (6F). A 4F sheath and wire were placed in the proximal superficial femoral artery as a fluoroscopic landmark to avoid arterial entry. Systemic heparin was maintained (activated clotting time of >250 seconds). Venography confirmed chronic occlusion of the left common femoral vein with reconstitution of the external iliac vein (Fig 1); a gooseneck snare was positioned from the jugular approach.

**Fig 2:**
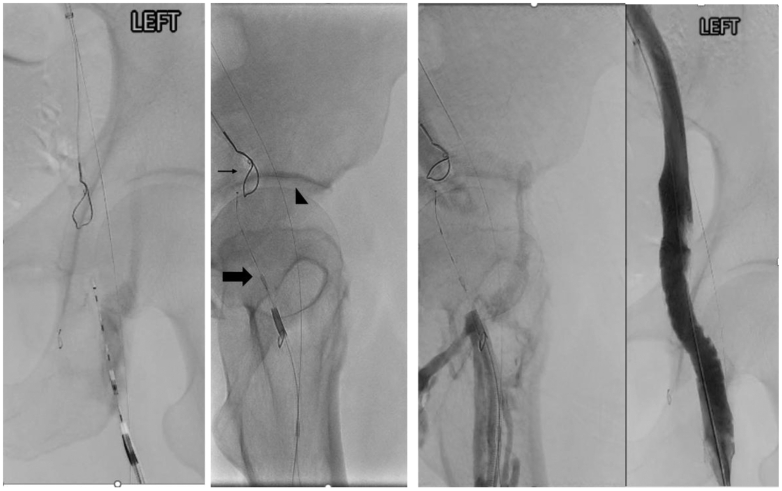
Recanalization of the chronic common femoral vein (CFV) occlusion using a Baylis radiofrequency (RF) wire while using different gantry angles (*small arrow*, a gooseneck wire from internal jugular access in the external iliac vein; *large arrow*, a Baylis wire crossing the CFV occlusion; *arrow head*, an 0.014-inch wire in the superficial femoral artery as a fluoroscopic landmark to avoid an inadvertent arterial injury. Final run after recanalization and stent placement.

Using a Cook TriForce catheter from the femoral approach, a Baylis curved RF wire was advanced under anterior-posterior, 30° left anterior oblique, and 30° right anterior oblique projections to ensure alignment with the snare (Fig 2). The wire tip was advanced in short, controlled increments with frequent multiplanar fluoroscopic rechecks to maintain the planned trajectory through scar tissue; the wire's energy penetration is approximately 1 mm.^5^ The wire traversed the occlusion, enabling through-and-through access with an Advantage Glidewire (Terumo). Serial venoplasty was performed up to 12-mm Mustang balloons (Boston Scientific), followed by high-pressure inflations (10-mm Conquest and 12-mm Atlas; BD Bard). Intravascular ultrasound (IVUS) examination confirmed an 11-mm external iliac diameter, appropriate landing zones, and no iliac compression. A 14 × 120-mm Abre stent (Medtronic) was deployed from the femoral confluence into the external iliac vein and postdilated to 14 mm. Completion venography/IVUS examination showed full expansion, brisk inline flow, and no residual collaterals.

**Fig 3:**
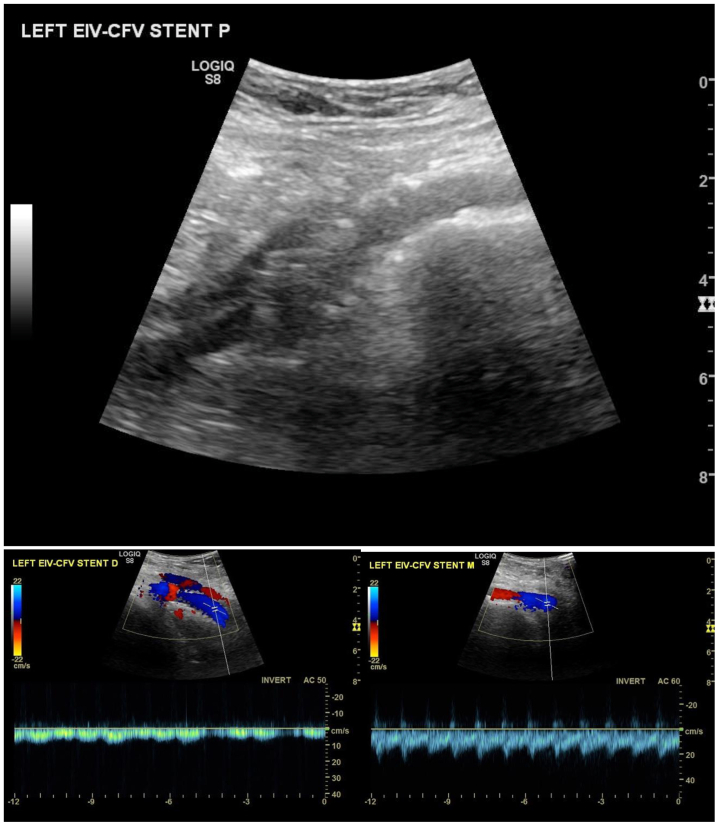
B-Mode and Color flow in left external iliac vein/common femoral vein (*EIV*/*CFV*) stent 3 months after placement.

At 4 months after recanalization, the patient reported complete resolution of venous claudication and pain with marked improvement in ankle swelling. No further interventions have been necessary. A 3-month duplex ultrasound examination (Fig 3) demonstrated a widely patent stent with normal color flow.

## Discussion

IVDU is a recognized risk factor for deep venous thrombosis and chronic venous occlusion via repetitive venous trauma, endothelial injury, and infection; femoral venous access (groin injecting) carries particularly high risk.^1^, ^2^, ^3^, ^4^ Crossing chronic fibrotic occlusions in the common femoral segment can be difficult; sharp techniques (eg, back end of a stiff Glidewire, Rösch-Uchida needle, or specialized crossing catheters) often fail. In the two prior failed attempts using sharp recanalization techniques, pushability was fully maximized; however, it ultimately was not the limiting factor. Those attempts demonstrated that the true barrier was the extreme density of the chronically scarred tissue in the groin. This experience informed our decision to use an RF wire in the current procedure, because RF energy relies on controlled thermal penetration rather than mechanical force and is therefore less dependent on pushability.

RF wires provide a salvage option when conventional and sharp strategies fail. Case series have reported feasibility and safety in refractory chronic venous occlusions—most commonly iliocaval—and in occluded venous stents^6^, ^7^, ^8^, ^9^ (Table). To our knowledge, no prior report has specifically described RF wire-assisted traversal of a chronic common femoral vein occlusion in a patient with a history of IVDU, as in this case. Restoring inline outflow with venoplasty and a dedicated venous stent resulted in early patency and symptomatic improvement at 3 months.

**Table:** Published reports of radiofrequency (*RF*) wire use for chronic venous occlusions

Reference	Anatomy/context	No. of patients/lesions	Technical success	Relevance
Keller et al, 2018^5^	Refractory chronic venous occlusions (primarily iliocaval)	18/20	High (single-center series)	RF wire feasible after failure of conventional/sharp crossing
Majdalany et al, 2018^6^	Chronically occluded venous stents (iliocaval)	15/15	High	RF can penetrate dense in-stent neointima
Shapiro et al, 2022^7^	Chronic iliocaval occlusion after failed prior therapy	10/10	60%	Demonstrates safety/efficacy as salvage in iliocaval setting

Technical considerations unique to the femoral region are critical. We used (1) multiplanar fluoroscopy/venography (anteroposterior, 30° left anterior oblique, 30° right anterior oblique) to ensure coaxial alignment, (2) a jugular-placed snare target to confirm trajectory, and (3) an arterial wire in a 4F superficial femoral artery sheath as a live landmark to avoid arterial engagement. These steps, together with IVUS examination for sizing/landing zones, mitigate the risk of injuring adjacent structures and help to ensure a controlled, intraluminal course.

Longer follow-up is needed to define durability after RF-assisted recanalization in the femoral segment and in patients with prior IVDU. Nevertheless, this case supports RF wire use as a rescue tool in carefully selected chronic femoral occlusions, provided meticulous technique is used to protect nearby neurovascular structures.

## Conclusions

This case illustrates the successful use of a RF wire for recanalization of a chronically occluded common femoral vein after failed sharp techniques. In patients with long-term sequelae of IVDU, the RF wire offers a safe and effective option for crossing resistant occlusions and enabling definitive stent reconstruction. This approach expands the therapeutic options for complex venous diseases and highlights the importance of individualized care in patients with challenging anatomy and histories.

## Funding

None.

## Disclosures

None.
